# Insights into Vestibular Migraine: Diagnostic Challenges, Differential Spectrum and Therapeutic Horizons

**DOI:** 10.3390/jcm14144828

**Published:** 2025-07-08

**Authors:** Marlon Cantillo-Martínez, Joan Lorente-Piera, Raquel Manrique-Huarte, Margarita Sánchez-del-Río, Nicolás Pérez-Fernández, Carlos Chico-Vila, David Moreno-Ajona, Pablo Irimia

**Affiliations:** 1 Department of Neurology, Hospital Occidente de Kennedy, Bogota 111611, Colombia; marlonxcantillom@gmail.com; 2 Department of Otorhinolaryngology, Clínica Universidad de Navarra, 31008 Pamplona, Spain; jlorentep@unav.es (J.L.-P.); rrmanrique@unav.es (R.M.-H.); nperezfer@unav.es (N.P.-F.); 3 Department of Neurology, Clínica Universidad de Navarra, 31008 Pamplona, Spain; msanchezd@unav.es; 4 School of Medicine, University of Navarra, 31009 Pamplona, Spain; 5 Headache Group, NIHR King’s Clinical Research Facility & SLaM Biomedical Research Centre, King’s College London, London SE5 9RS, UK; david.1.moreno_ajona@kcl.ac.uk; 6 Wolfson SPaRC, Institute of Psychiatry, Psychology and Neuroscience, King’s College London, London SE5 9PJ, UK

**Keywords:** vestibular migraine, vertigo, dizziness, migraine, pathophysiology

## Abstract

Vestibular migraine (VM) commonly causes recurrent vertigo, but diagnosing and managing it can be difficult due to symptom overlap with other vestibular and headache disorders. This review provides a comprehensive update on VM, beginning with the diagnostic criteria established by the International Headache Society and the Bárány Society, who have increased awareness of this condition. While the pathophysiology is not yet completely understood, there is evidence of a complex interaction between the nociceptive and vestibular systems. Treatment approaches are primarily empirical and lack robust, high-quality evidence. Often, antihistamines and benzodiazepines are used for quick symptom relief, while the efficacy of triptans is still uncertain. Preventive measures include lifestyle changes, vestibular rehabilitation, oral migraine prophylactics, Botulinum toxin type A, and, more recently, CGRP-targeted therapies. Due to diagnostic uncertainties and the absence of standardised treatment protocols, further research—particularly randomised controlled trials—is crucial for establishing evidence-based guidelines.

## 1. Introduction

The link between migraine and vertigo was identified in the last century, first noted in children and then later in adults [1]. Epidemiological studies have demonstrated that the coexistence of migraines and vertigo occurs approximately three times more frequently than would be expected by chance, affecting the quality of life of patients [2,3]. This evidence suggests that vertigo episodes could have a pathophysiological connection with migraine, leading to the term vestibular migraine (VM) [4]. One of the earliest descriptions of this association was provided by Kayan and Hood in 1984, who reported central vestibular abnormalities in patients with migraine, laying the groundwork for what is now conceptualised as VM [5]. Over the following decades, this link gained increasing attention, ultimately leading to the progressive adoption of the term VM in the literature [6]. However, VM was not formally recognised as a clinical entity until 2012, when a joint consensus document by the International Headache Society and the Bárány Society proposed standardised diagnostic criteria [7]. This publication reinforced the conceptual and mechanistic link between migraine and vestibular dysfunction and facilitated a more consistent framework for research and clinical diagnosis. Nonetheless, ongoing debate remains regarding whether VM constitutes a single, well-defined disorder or represents a spectrum of phenotypes with shared features but potentially distinct pathophysiological mechanisms, clinical profiles, and therapeutic responses. As a result, this condition is frequently misdiagnosed [8,9]. Consequently, gaining a deeper understanding of VM is crucial for enhancing diagnostic precision and optimising treatment results [10].

## 2. Methodology

A non-systematic bibliographic search was conducted in the PubMed/MEDLINE database, starting in 1996. The search utilised terms like ‘VM’, ‘vertigo’, ‘pathophysiology’, ‘differential diagnosis,’ and ‘treatment.’ The authors independently selected articles based on their inherent characteristics and relevance to the topic. Both English and Spanish publications were included, while records unrelated to the subject, lacking conclusive data or falling outside the scope of the study were excluded. The results were analysed and categorised according to themes, considering the conceptual complexity of the subject.

## 3. Epidemiology

VM is one of the most common causes of episodic vertigo in both adults and children [11]. Population-based studies have reported a prevalence ranging from 1% to 2.7% within the general population, with an age of onset, in most cases, between 40 and 50 years [2,12]. In vertigo clinics, it accounts for 6–11% of cases, whereas in migraine clinics, the prevalence rises to 9–13% of patients [13,14,15,16]. Given the prevalence of this disease, it is important to note that VM is considered the second most common vestibular disorder after benign paroxysmal positional vertigo. This condition primarily impacts women, typically starting around the age of 40. Furthermore, VM can worsen during menstruation, a trend frequently seen in other migraine subtypes [17,18].

## 4. Pathophysiology

The pathophysiology of vestibular migraine remains incompletely understood. Proposed mechanisms include the involvement of neuropeptides related to pain transmission, vascular and inflammatory pathways, genetic susceptibility, and functional alterations in brain networks [19]. The condition is thought to arise from interactions between the nociceptive and vestibular systems [20,21,22].

Different studies suggest that genetic factors play a role in VM, as it appears to have a familial aspect, with certain instances showing an autosomal dominant inheritance pattern coupled with moderate-to-high penetrance [23]. However, other investigations indicate that the available evidence supporting heritability in VM is limited [24]. Thus far, candidate gene studies have not identified any causal mutations, suggesting that, like other migraine subtypes, VM may be a polygenic disorder.

Regarding headache pathways, the trigeminovascular reflex is activated at the level of the meningeal nociceptors of the trigeminal nerve, leading to the release of neuropeptides such as CGRP [25]. Evidence suggests that trigeminal fibres also innervate the labyrinthine vessels and the inner ear [26], and that CGRP is present in the vestibular sensory epithelium of the membranous labyrinth as well as in the vestibular nuclei [27,28]. CGRP released in the peripheral vestibular system affects the dynamics of the otolithic organs [29], resulting in variations in afferent responses that may contribute to motion sickness [30]. Furthermore, experimental studies in animal models demonstrate plasma extravasation and the release of inflammatory mediators and neuropeptides in the inner ear, specifically in the apical spiral ganglion, the modiolus, and the intralabyrinthine areas of the superior and inferior vestibular nerves, following the administration of serotonin, a crucial neurotransmitter in the pathophysiology of migraine [31]. These findings support the idea of a functional interaction between the peripheral trigeminal and vestibulocochlear systems, known as the trigemino-vestibulocochlear reflex, which could play a role in the pathophysiology of migraine and the development of associated symptoms, such as vertigo and sound sensitivity [32]

Once peripheral afferents are activated, nociceptive signals are relayed to the brainstem, targeting the spinal trigeminal nucleus [25]. This nucleus has reciprocal connections with the vestibular nuclei [33,34] and research suggests that the vestibular nuclei possess receptors that can be sensitised by CGRP [35,36]. Beyond these direct links, the spinal trigeminal nucleus also interacts indirectly with the vestibular nuclei through nociceptive modulatory structures such as the parabrachial nucleus, the raphe nuclei and the locus coeruleus [37,38]. These pathways may play a role in the vestibular symptoms observed in migraine patients [32].

Given its potential involvement in the pathophysiology of VM, CGRP has been proposed as a candidate biomarker for this condition. Nonetheless, a recent study assessing interictal CGRP levels in both the plasma and saliva of patients with VM and episodic migraine found no evidence supporting its diagnostic utility [39]. It is important to consider that plasma CGRP levels may not accurately reflect local levels within vestibular pathways, which constitutes a significant limitation when evaluating its reliability as a biomarker for VM. Therefore, further research is warranted to elucidate the precise role of CGRP in migraine-associated vestibular symptoms [39].

Next, nociceptive information is transmitted to subcortical structures, especially the ventral posteromedial thalamic nucleus [25]. This area, together with the ventral posterolateral thalamic nucleus, plays a role in processing vestibular information. Thus, the thalamus functions as a multisensory integration centre where vestibular and nociceptive inputs come together [40]. Notably, neuroimaging studies have demonstrated that patients with VM exhibit significantly greater thalamic activation compared with individuals with migraine without aura and healthy controls [41], reinforcing the thalamus’s role in the multimodal sensory sensitisation observed in VM.

Ultimately, nociceptive information reaches the cortical level, where the posterior insular-opercular area is proposed to be the primary nociceptive cortex. This area also functions as a convergence zone with the vestibular pathway. Disruptions in functional connectivity between the sensorimotor network and the cortical vestibular network could lead to increased sensitivity in vestibular sensory processing [42,43,44]

Moreover, brain imaging studies in patients with VM have demonstrated increased metabolism in the parietoinsular areas and both thalami, further supporting the activation of the vestibulothalamocortical pathways [45]. Collectively, these findings suggest that VM may represent a point of convergence between the pathophysiological mechanisms of migraine and vestibular dysfunction [46].

**Figure 1:**
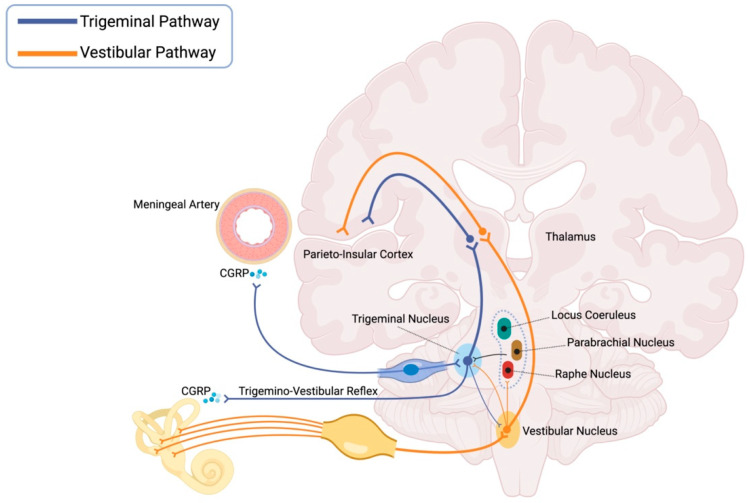
Vestibular migraine pathophysiology. Connections between the trigemino-nociceptive pathway (in blue) and the vestibular pathway (in orange) include, at the peripheral level, trigeminal innervation of the labyrinthine vessels and inner ear, where CGRP is released, and a correlated trigemino-vestibulocochlear reflex. In the brainstem, both direct and indirect connections exist between the spinal trigeminal nucleus and the vestibular nuclei, which also express CGRP. At the central level, further connections have been identified in the thalamus and parieto-insular cortex. Taken together, these findings highlight the close functional relationship between the trigeminal and vestibular systems and help explain the association between vestibular symptoms and headache observed in vestibular migraine. Created with BioRender.com.

In summary, the pathophysiological hypothesis for VM includes the integration of trigeminal nociceptive inputs with the vestibular system, the role of CGRP, and the abnormal sensitisation of central structures in the brainstem, thalamus and cortex (Figure 1) [32,47,48,49].

## 5. Diagnostic Criteria

VM is a complex and heterogeneous condition at the interface of neurology and otology. Consensus diagnostic criteria were first jointly established by the Bárány Society and the International Headache Society in 2012 [7], and these were later included in the third edition of the International Classification of Headache Disorders (ICHD-3) in 2018 [50]. In 2022, Lempert and colleagues published an updated review, which maintained the original diagnostic criteria unchanged but added relevant developments [51]. One of the main updates was the separate publication of diagnostic criteria for paediatric vertigo syndromes associated with migraine, acknowledging the age-dependent variability of the disorder.

Despite these efforts, the current classification system only recognises “definite VM” under ICHD-3 and “probable VM” according to the Bárány criteria. This narrow dichotomy has been criticised for failing to encompass the full clinical spectrum of VM, leaving many patients unclassified despite having migrainous vertigo symptoms and responding to migraine-specific therapies. Recent research has explored the possibility that VM is not a single, uniform entity but rather a cluster of phenotypes with potentially distinct underlying mechanisms. Emerging evidence from cluster analyses supports this view, suggesting the existence of subtypes based on clinical presentation, attack duration, response to treatment, and associated symptoms, which may reflect different pathophysiological pathways. The diagnostic criteria, as outlined by Lempert et al., are presented in Table 1A,B [51]

The vestibular symptoms shown Table 1 and considered for diagnosis include the following:

- **Spontaneous vertigo**, which encompasses internal vertigo (false self-motion perception) and external vertigo (false sensation of the visual environment spinning or flowing). It is the most frequently reported vestibular symptom, with an estimated prevalence of around 67% in the context of VM [2].
- **Positional vertigo**, which occurs when changes in head position occur.
- **Visually induced vertigo**, which is triggered by complex or large-scale visual motion stimuli.
- **Head motion-induced vertigo**, which is dizziness elicited during head movements.
- **Head motion-induced dizziness with nausea**, which is characterised by disturbed spatial orientation.

This strict classification risks excluding patients with atypical or overlapping phenotypes from diagnosis [52]. For example, dizziness qualifies as a symptom only when triggered by head movement and accompanied by typical migraine features, thereby excluding manifestations such as spatial disorientation or visual motion sensitivity—common in conditions like Mal de Débarquement Syndrome or Persistent Postural-Perceptual Dizziness (PPPD), both of which are prevalent among migraine sufferers. Additionally, the rigid duration criteria (5 min to 72 h) fail to accommodate the brief or extended episodes that many patients experience, especially during the prodromal or residual phases of a migraine attack [53].

Moreover, the timing relationship between migraines and vestibular symptoms has been significantly neglected in research—an essential aspect that warrants further investigation [51]. Epidemiological studies indicate that migraine typically arises 5–10 years prior to the onset of vertigo, with vestibular symptoms generally emerging around the fifth decade of life [54,55]. It is crucial to ascertain whether vestibular issues manifest before, alongside or after headache symptoms to distinguish primary VM from simultaneous vestibular disorders or secondary migraine features. Our research emphasises the importance of monitoring the progression of vestibular and auditory symptoms in conjunction with the onset of migraines, examining their sequence of appearance, duration, treatment responses and potential phenotypic convergence time.

Furthermore, there have been proposals to expand the diagnostic framework to include non-vertiginous symptoms, such as fluctuating hearing loss. This is intended to cater to a broader patient population, particularly when vertigo is not the main issue [56,57]. However, broadening the criteria might unintentionally bring other vestibular disorders into the category of VM [58]. Although vertigo can be considered a component of a migraine aura, the duration of VM episodes—which may last from seconds to days—does not correspond with the typical aura duration of 5–60 min [59]. These subtleties have generated debates during the formulation of the criteria, especially regarding the balance between sensitivity and specificity.

The lack of universally recognised endophenotypes or subtypes in VM complicates both diagnosis and treatment. Certain patients exhibit significant migrainous features during vertigo episodes, whereas others might present solely with vestibular symptoms, having only a remote migraine history [60]. This situation has led some researchers to suggest a spectrum model that covers both central and peripheral symptoms. This model includes cases that respond to migraine preventive treatments, even if they qualify for Menière’s disease or benign paroxysmal positional vertigo. These overlaps are important because migraine-related mechanisms, especially those related to the trigemino-vascular system, have been linked to inner ear dysfunction, cochlear vasospasm, and/or otolithic hypersensitivity [9].

Psychiatric comorbidities, particularly anxiety and somatoform disorders, are often overlooked in VM, even though they are common and can complicate the clinical picture [61]. Recognising these conditions is crucial for both accurate diagnosis and effective treatment strategies, as these patients typically require comprehensive care that extends beyond medication alone [62].

These limitations underscore the a priori nature of existing VM definitions, which might overlook valid clinical variants. Instead of adhering to a rigid nosological boundary, a phenotype-focused strategy—considering auditory symptoms, psychiatric comorbidities, and variability in duration and chronological profile—may be necessary to enhance the diagnosis and more accurately reflect clinical reality. Supporting this approach requires precise phenotypic definitions, rigorous exclusion of confounding variables, and comprehensive longitudinal documentation. Such a methodology can provide a robust foundation for redefining diagnostic thresholds and guiding future updates to classification systems.

## 6. Clinical Presentation, Physical Examination and Audiovestibular Test Results

In most cases of VM, neurological and neuro-otological evaluations seem normal during asymptomatic periods; however, nonspecific signs of vestibular involvement may be detected in the interictal phase [63]. During symptomatic episodes, slight abnormalities have been described, including various types of nystagmus—such as gaze-evoked nystagmus, saccadic pursuit, bidirectional, central positional nystagmus and horizontal or vertical spontaneous nystagmus—as well as minor deficits in the vestibular-ocular response [64]. Regardless of these findings, diagnosis of VM is clinical.

A comprehensive bedside vestibular evaluation is essential for every patient with suspected VM. This evaluation should include examination of ocular motility, spontaneous and positional nystagmus, vestibulo-ocular reflexes, and postural stability using gait and balance tests. Depending on the predominant clinical phenotype (whether the vertigo is spontaneous, positional, or visually induced), certain complementary studies may be included, such as vestibular tests that provide information on different parts of the vestibular system: the caloric test, which evaluates the horizontal semicircular canal at low frequencies; the video head impulse test (vHIT), which evaluates all semicircular canals at high frequencies; and vestibular-evoked myogenic potentials (VEMPs), which explore the function of the otolithic organs, as follows: cervical (cVEMPs) for the saccule and inferior vestibular nerve, and ocular (oVEMPs) for the utricle and superior vestibular nerve [65]. This vestibular testing can identify additional findings, such as uncompensated vestibular hypofunction or positional nystagmus with central features [66]. Some VM patients exhibit caloric response asymmetries and oculomotor deficits, these may indicate preexisting or comorbid vestibular problems, though whether this could have triggered increased vestibular symptoms is unknown. Typically, VM sufferers frequently report heightened motion sensitivity and are more prone to symptom aggravation following vestibular assessments, particularly after caloric tests, and it is important to note that the tendency of nystagmus assessed during the caloric test by the directional preponderance (DP) parameter in central vestibular lesions (such as VM) may be less reliable than in peripheral lesions [67]. It is important to note that, in patients with VM, objective vestibular test results often lack correlation with each other or with clinical severity. Consequently, no single test is sufficient for diagnosis, and results should be interpreted in combination to obtain a more comprehensive understanding of vestibular function. VEMPs are valuable for distinguishing VM from Ménière’s disease (MD), despite variable results [64]. A preserved oVEMP or cVEMP response amid overlapping symptoms can assist in ruling out MD, with some studies indicating a high negative predictive value. While isolated cVEMPs alone cannot confirm a diagnosis, their incorporation into comprehensive diagnostic algorithms has been proposed and is under active investigation [68].

Audiology evaluations are also necessary, including audiometric evaluations that can offer useful insights. Although cochlear symptoms are not included in the current diagnostic criteria for VM, they are increasingly recognized as relevant clinical features that may complicate differential diagnosis. A subset of patients with VM report transient or fluctuating sensorineural hearing loss, typically in the low-frequency range, which is usually mild and reversible [69]. Additionally, other auditory assessments such as otoacoustic emissions (OAEs) and auditory brainstem responses (ABRs) may reveal subtle or nonspecific abnormalities in individuals with chronic migraine, even when pure-tone thresholds are within normal limits [70]. Although these tests lack diagnostic specificity for vestibular migraine, they offer valuable insights beyond conventional pure-tone audiometry. OAEs provide objective information about outer hair cell function in the cochlea, potentially revealing early cochlear dysfunction that is undetectable by audiometric evaluations. Similarly, ABRs assess neural conduction along the auditory pathway and may reflect central auditory processing alterations, as observed in some migraine patients [71]. Together, these objective measures help capture both peripheral and central auditory involvement, contributing to a more comprehensive functional assessment in suspected cases.

## 7. Differential Diagnosis of Vestibular Migraine: Clinical Approach

Currently, diagnosis depends largely on a comprehensive clinical history, focusing on the characteristics of the attacks—such as their frequency, duration, triggers and related symptoms. Importantly, as mentioned earlier, it is crucial to document the long-term timeline of symptom progression to differentiate VM from other similar vestibular disorders and to determine if there is a consistent temporal relationship between the vestibular symptoms and migraine features [72].

A significant challenge in these patients is the overdependence on the qualitative symptoms described. Words such as dizziness, vertigo, lightheadedness, unsteadiness and presyncope are often used inconsistently by both patients and healthcare providers, leading to confusion and misinterpretation. Traditional methods that have attempted to categorise dizziness types based solely on symptom quality are now considered unreliable and outdated. For example, the subjective feeling of spinning or external vertigo does not necessarily signify a vestibular issue, just as feeling faint does not rule one out. Furthermore, research indicates that patients’ accounts of dizziness symptoms can vary greatly between different evaluations and are not consistently reproducible [73,74].

Current clinical practice emphasises a structured approach to history-taking, which includes a detailed analysis of episode timing, potential triggers, associated symptoms and the evolution of symptoms over time. While traditional definitions—vertigo as a false sensation of movement, dizziness as a disturbance of spatial orientation and unsteadiness as a vague sense of imbalance [75]—are useful for clinical communication, they are insufficient to determine the underlying aetiology.

VM is usually marked by repeated vestibular symptoms, including vertigo, dizziness or imbalance, which occur alongside migraine canonical symptoms like migrainous headache (approximately 50% of attacks), photophobia, phonophobia, visual aura, nausea and some non-canonical like osmophobia [76].

However, none of these additional symptoms are uniquely indicative of VM. The only characteristic that shows moderate sensitivity is the exacerbation of symptoms with head movement or during motion, illustrating the inherent hypersensitivity of the vestibular system [71]. The absence of a clear marker highlights the importance of considering the quality of symptoms in conjunction with their timing and relationship to migraine history or phenotype, underscoring the challenge of making a diagnosis based solely on isolated symptoms.

VM is an episodic vestibular disorder that frequently shares symptoms with other neurotological conditions, leading to potential misdiagnoses, Notably, migraine headaches are absent in up to 50% of VM attacks, which complicates clinical identification, particularly in patients without a prior history of migraine. Therefore, clearly defined semiological features beyond headache—such as photophobia, phonophobia, visual aura, or motion sensitivity—are essential for achieving an accurate diagnosis [77].

### 7.1. Benign Paroxysmal Positional Vertigo (BPPV)

BPPV represents 8–15% of individuals experiencing moderate-to-severe vertigo [78]. Interestingly, almost 50% of these individuals may have a history of migraine [79] and migraine sufferers appear to have a greater likelihood of developing BPPV, with prevalence rates as high as 12.3% [80,81]. Although the precise link between these two conditions remains unclear, one theory suggests that migraine-related vascular alterations may impair microvascular flow to the inner ear, leading to damage in vestibular hair cells and the dislodgment of otoconia from the macula. This proposed mechanism may explain why some patients with chronic otolith dysfunction experience improvements with vasodilator therapy [82].

It is crucial to understand that having both migraine and BPPV does not imply they have a common cause or that one is a type of VM, as even BPPV can be secondary. Although these conditions often coexist, they typically occur independently of each other regarding their timing. A diagnosis of VM can only be established when vestibular episodes closely align with migraine symptoms and fulfil recurrence criteria, which usually stipulate five or more attacks. If a patient suffers from migraine alongside recurrent BPPV, it should be recognised as a comorbidity, not a direct diagnostic link overlap.

Additionally, patients with BPPV may suffer from various types of headaches, either during the interictal phase or as lingering symptoms after vertigo episodes [83]. These headaches often do not align with the migraine profile, highlighting the need for continuous phenotyping and chronological analysis to differentiate VM from closely related vestibular disorders. Tension-type headaches are most observed, followed by migraines and cervicogenic headaches. These headaches appear to be independently associated with BPPV and can exacerbate patient distress [84].

Clinically, vertigo linked to BPPV often resolves within a minute and can be triggered by specific head movements. A key diagnostic characteristic is the presence of positional nystagmus, which is generally fatigable and predominantly affects one of the semicircular canals. In contrast, the positional nystagmus related to VM is usually more persistent, atypical and not confined to a single canal [85].

### 7.2. Menière’s Disease (MD)

MD is a condition found in 3–11% of ENT consultations [86]. It affects the inner ear and is characterised by unexpected bouts of vertigo, often accompanied by fluctuating, usually unilateral, sensorineural hearing loss, tinnitus and a sensation of fullness in the ear [87]. Histologically, it is associated with endolymphatic hydrops, which is the abnormal expansion of the endolymphatic spaces in the labyrinth [88]. However, the exact connection between hydrops and the clinical syndrome remains unclear. In addition, up to five distinct phenotypes are now recognised, which may aid in a more precise characterisation [89].

In MD, vertigo attacks typically begin with cochlear symptoms, such as roaring tinnitus, aural pressure and muffled hearing. Vertigo typically develops within minutes to hours, quickly reaching its peak intensity and lasting anywhere from 20 min to 12 h, with an average duration of 2–3 h. Horizontal spontaneous nystagmus is often observed during these episodes, initially beating towards the affected ear in the excitatory phase and then reversing direction within 12 h as it moves into the inhibitory phase [73]. Additionally, a recovery nystagmus phase may also be recorded, while the use of a head impulse test or a Halmagyi pathological manoeuvre have also been reported [90].

Over time, the variable sensorineural hearing loss associated with MD usually progresses and stabilises at more severe levels. Initially, this hearing loss mainly affects low frequencies (250, 500 and 1000 Hz). This pattern is associated with the cochlea’s apical region, which is responsible for processing low-frequency sounds and is susceptible to pressure changes caused by endolymphatic hydrops [91]. As the disease progresses, the audiometric curve becomes progressively flatter, reflecting the involvement of mid- and high-frequency ranges and resulting in pancochlear sensorineural hearing loss. Tinnitus often becomes a persistent condition, with bilateral involvement occurring in approximately 30% of patients as the condition progresses.

Even more, the differential diagnosis between MD and VM can be challenging, as both conditions may present with overlapping symptoms. MD is associated with a notably high prevalence of headache [92], and approximately one-quarter of patients with VM also present with cochlear symptoms [93]. This association may be explained by the connections between the trigeminovascular and vestibular systems at the auditory level [94]. A further diagnostic clue lies in the dissociation frequently observed between vestibular test results: while video head impulse testing (vHIT) is often normal in MD, caloric irrigation and the skull vibration-induced nystagmus test (SVINT) typically reveal unilateral vestibular hypofunction, which contrasts with the findings in VM. In this context, Murofushi et al. have also proposed the concept of a *MD–VM overlapping syndrome,* reflecting patients who meet partial criteria for both disorders and may represent a continuum rather than distinct entities [95].

In recent years, magnetic resonance imaging (MRI) techniques capable of visualising endolymphatic hydrops after intravenous or intratympanic administration of gadolinium have been developed. MRI can detect enlargement of the endolymphatic spaces, aiding in the diagnosis of MD [96]. However, studies have shown that approximately 20–30% of patients with VM may also exhibit signs of endolymphatic hydrops on MRI [97], which is why the diagnosis of VM remains fundamentally clinical. In patients with VM it is more common to find white matter hyperintensities in magnetic resonance imaging.

Recent studies have identified distinct proinflammatory cytokine profiles in VM compared with MD [98]. Biomarkers such as IL-1β, IFN-γ, CCL3, CCL22 and CXCL1—detectable through peripheral blood sampling—may, in the future, serve as valuable aids for differential diagnosis and personalised treatment approaches.

Furthermore, distinguishing MD from VM hinges on a thorough evaluation of audiometric patterns. Unlike the MD hearing loss pattern noted in VM, when it occurs, it typically remains non-progressive, affects both ears and lacks a distinctive pattern, often impacting high frequencies above 2000 Hz [89]. Additionally, the presence of distinctive and well-recorded cochlear symptoms and their progression over time significantly support the diagnosis of MD. Despite advances in complementary testing, the diagnosis of MD remains primarily clinical. Finally, although both conditions can cause episodes of vertigo, the association with migraine-like features, such as headaches, photophobia, or visual aura, is a defining feature of VM and is generally not seen in MD.

### 7.3. Stroke and Vertebrobasilar Transient Ischaemic Attacks (TIA)

Cerebrovascular disorders are estimated to account for approximately 3–7% of cases presenting with vertigo [99], with the majority involving vertebrobasilar circulation. Vascular compromise in this territory can produce a wide range of symptoms, including vertigo, headache, dysarthria, paresthesias, confusion and amnesia [100]. While focal neurological signs are a crucial clinical indicator of a vascular cause, it is vital to realise that isolated vertigo can sometimes be the only sign of a stroke, especially if the labyrinthine artery is involved [101]. Generally, when vertigo appears in older patients—particularly those with known cardiovascular risk factors—it raises strong suspicions of vascular causes, particularly if the onset is sudden or if there are signs of vertebral or proximal basilar artery issues. In these instances, obtaining brain MRI is critical for achieving an accurate differential diagnosis [51,102].

MRI is significantly more sensitive than CT for detecting posterior fossa and brainstem lesions, which are often implicated in central vestibular syndromes. Despite this, suboptimal practices still persist in routine clinical settings, where brain CT is frequently overused despite its limited diagnostic yield in this context. In the differential diagnosis, vestibular neuritis must also be considered, as it can present with acute prolonged vertigo mimicking posterior circulation stroke, especially in the so-called neuritis-like presentations [103].

### 7.4. Vestibular Paroxysmia

Vestibular paroxysmia is a clinical syndrome characterised by brief, recurring episodes of vertigo, typically lasting from a few seconds to one minute and occurring several times a day. This condition is considered rare, with an estimated prevalence of approximately 1–4 cases per 100,000 individuals. The proposed underlying mechanism involves ephaptic transmission along the vestibulocochlear nerve, often caused by microvascular compression at the nerve root entry point, most often caused by the AICA [104].

Clinically, patients commonly experience episodes of stereotyped vertigo, which may be accompanied by temporary oscillopsia or auditory symptoms, such as tinnitus or hyperacusis [105]. These episodes often occur spontaneously but can also be triggered by head movements, loud noises or hyperventilation. Despite this, neurological and vestibular examinations typically appear normal between episodes, although a residual headache may persist in nearly half of the cases [106].

High-resolution MRI utilising CISS or FIESTA sequences can identify vascular compression with the eighth cranial nerve; however, these findings lack specificity, as similar findings occur in many asymptomatic individuals [106]. The diagnosis primarily depends on the clinical presentation, particularly the brief duration and high frequency of episodes, as well as a positive response to carbamazepine or oxcarbazepine [104]. The extremely brief and patterned nature of these episodes facilitates differentiation between vestibular paroxysmia and VM, as well as other vestibular conditions.

### 7.5. Otic Capsule Dehiscences (OCD)

OCD, categorised within the third mobile window syndrome, involves structural abnormalities in the bony labyrinth that interfere with the inner ear’s fluid dynamics [107]. Its actual prevalence is unclear and likely underestimated, often due to misdiagnosis with other vestibulocochlear disorders. While superior semicircular canal dehiscence (SSCD) is the most frequently recognised variety, other areas, such as the cochlear–facial and cochlear–carotid interfaces, may also be involved [108].

Clinically, individuals may exhibit symptoms such as vertigo, imbalance, hearing impairment, headache between episodes, tinnitus and often, though not definitively, autophony and/or heightened sensitivity to sound or pressure stimuli, which may present as Tullio or Hennebert phenomena [109]. Audiologically, third window syndromes typically present with low-frequency conductive or mixed hearing loss, often with a fluctuating course, which may mimic the audiometric profile of MD [110]. Notably, specific dehiscences have been linked to varying auditory symptoms and radiological indicators of endolymphatic hydrops, especially when situated outside the usual superior canal region [111].

These abnormalities can lead to significant disruptions in homeostasis within the inner ear, altering the endolymph pressure gradients and affecting the fluid dynamics of the labyrinth. VEMP tests often reveal distinct changes, such as increased ocular VEMP amplitudes at high frequencies, particularly in cases of SSCD. This is in contrast to migrainous attacks, where the interaural asymmetry index might be raised due to a decrease in amplitude [112].

High-resolution computed tomography plays a crucial role in accurately identifying these defects, while MRI can help reveal secondary changes, such as hydrops, as initially reported by our group in 2024 [113]. Identifying OCD is vital to avoid misdiagnosis, especially in patients who exhibit overlapping vestibular and auditory symptoms.

### 7.6. PPPD or 3PD

While PPPD is categorised as a chronic vestibular syndrome rather than as a condition marked by recurrent spontaneous vertigo, its high occurrence and frequent overlap with VM (15–30% overlap) justify its inclusion in the differential diagnosis [114]. PPPD is characterised by ongoing dizziness, which may be accompanied by headaches or other nonspecific vestibular symptoms, non-spinning vertigo and a sense of unsteadiness lasting more than 3 months. These symptoms are typically worsened by upright posture, active or passive movement or exposure to complex visual settings [115].

In 2017, the Bárány Society established diagnostic criteria for PPPD, consolidating previous labels such as chronic subjective dizziness, phobic postural vertigo, space and motion discomfort and visual vertigo [116]. For diagnosis, symptoms must result in notable distress or functional impairment and should not be more accurately attributed to a different medical condition, though PPPD can occur alongside other vestibular or neurological disorders that initially caused it.

PPPD can arise from various acute vestibular, neurological, cardiac or psychological events such as vestibular neuritis, concussion, autonomic dysfunction, panic attacks or migraine episodes [117]. Significantly, PPPD symptoms may continue even after the initial triggering event has resolved independently.

The disorder indicates a maladaptive response within the balance processing systems, resulting in a continuous dependence on visual and cognitive strategies over vestibular inputs [115]. It is essential to identify PPPD, especially in patients experiencing chronic symptoms post-acute vertigo, as prompt recognition can enable effective and targeted multidisciplinary treatment management. Given the symptom overlap, more studies are warranted to understand if PPPD is distinct from a potentially chronic form of VM or if both disorders constitute part of the same spectrum. A summary of the different findings in the entities comprising the differential diagnosis is shown in Table 2.

### 7.7. Treatment

Follow-up studies on patients with VM indicate that, although the frequency and intensity of headache and vertigo episodes tend to diminish over time, only a small percentage experience complete resolution without treatment [118]. Consequently, the management of VM should involve both acute interventions and preventive measures. Nevertheless, a definitive treatment algorithm remains unestablished [119]. Currently, treatment recommendations mainly stem from observational studies, anecdotal clinical experience and expert consensus. Moreover, systematic reviews have repeatedly emphasised the absence of well-designed randomised controlled trials, which hampers the formulation of specific, evidence-based treatment guidelines for this condition [120]. Indeed, recent influential reviews have highlighted the pressing requirement for practical, condition-specific management strategies for VM, especially considering the overall poor quality and variability of existing evidence [121]. Thus, the current recommendations are based on an integrative approach that combines the limited evidence available, adjusts established migraine management protocols and includes widely accepted therapies for vertigo treatment [120].

#### 7.7.1. Acute Treatment

At present, research specifically examining the acute treatment of VM is limited. Most existing evidence centres around the use of triptans. For example, a retrospective survey indicated that sumatriptan improved vertigo in migraine patients [122]. In a similar vein, a retrospective study of 18 patients has demonstrated that almotriptan effectively and safely alleviated both vertigo and headaches in those with VM [123]. Furthermore, studies involving 25 migraine patients revealed that rizatriptan could reduce motion sickness induced by vestibular stimuli [124]. However, these findings are countered by recent clinical evidence, such as a controlled study with 17 patients that compared zolmitriptan 2.5 mg to a placebo, which showed no significant benefits [125]. Additionally, a double-blind, randomised trial of rizatriptan 10 mg versus a placebo with 222 participants indicated that rizatriptan was ineffective after 1 h for VM attacks and had minimal impact on symptoms at the 24 h mark [126]. Taken together, this evidence aligns with clinical experience, suggesting that triptans may be less effective for acute episodes of vertigo compared with migraine headache attacks [121]. Dizziness is indeed included as a potential side effect for triptans [122].

A meta-analysis of 17 clinical trials assessing the effectiveness of antihistamines (like dimenhydrinate 50 mg and cinarizine 25–50 mg) versus benzodiazepines (diazepam 5 mg IV and lorazepam 2 mg IV) for treating acute vertigo found that antihistamines offered significantly better symptom relief [127]. Moreover, patients experiencing severe and prolonged acute attacks may benefit from inpatient management, including intravenous hydration and antiemetics (promethazine 25 mg and metoclopramide 10 mg) [128]. Significantly, reports indicate clinical improvement in severe VM cases lasting over 1 day after intravenous administration of 1000 mg methylprednisolone [129]. While sulpiride is sometimes prescribed to treat migraine-related symptoms such as vertigo and anxiety, clinical evidence does not support its use for VM [130]. In conclusion, although there is insufficient high-quality evidence for acute treatment, a combined approach seems justifiable—utilising effective headache medications, such as NSAIDs and triptans, in conjunction with treatments aimed at relieving vertigo symptoms, including antihistamines.

The role of anti-CGRP therapies should be considered in the acute treatment of VM and warrants further exploration.

#### 7.7.2. Preventive Treatment

##### Nonpharmacological Interventions

Due to the lack of robust data, recent authors have suggested a practical, evidence-informed management strategy that combines clinical experience with existing studies rather than relying exclusively on systematic meta-analyses [121]. Similar to general migraine management, the preventive approach for VM should encompass both pharmacological and nonpharmacological methods. A 2023 Cochrane review evaluated nonpharmacological lifestyle changes and found that the evidence supporting these interventions for VM is still limited [131]. Nevertheless, lifestyle modifications are consistently highlighted [132] and general migraine management recommendations can be applied. These include reducing stress, identifying and avoiding triggers, maintaining good sleep hygiene, avoiding fasting, dehydration, caffeine and alcohol, keeping a headache diary, engaging in consistent physical activity and following a healthy body routine [133]

##### Vestibular Rehabilitation

Moreover, vestibular rehabilitation can be incorporated into the nonpharmacological strategy [134]. Rehabilitation strategies often encompass dynamic balance training, gaze stabilisation exercises and head–eye coordination activities. For individuals experiencing visual vertigo, optokinetic stimuli may be incorporated to lessen hypersensitivity to visual motion. It is common to experience a temporary increase in symptoms while performing these exercises, which indicates the usual adaptation process [135]. Alghadir and Anwer highlight that structured vestibular rehabilitation programmes, which include Cawthorne–Cooksey exercises, gaze stabilisation, habituation tasks and postural control activities, have demonstrated significant benefits in alleviating dizziness and disability among patients with VM [136]. Their research showed that a 6-week programme focused on progressive eye–head movement coordination, standing balance tasks on both firm and compliant surfaces, as well as dynamic gait training significantly improved scores on the Dizziness Handicap Inventory and enhanced postural stability. This approach also integrated weekly supervised sessions with daily home-based exercises, highlighting the importance of gradual sensory reweighting and neural adaptation compensation.

#### 7.7.3. Pharmacological Treatment

For preventive drug treatment, as with other migraine types, it is advised when vestibular migraine episodes occur multiple times each month, last for several weeks or greatly affect the patient’s daily life, even if not all instances meet the VM criteria [137]. Retrospective studies suggest that, overall, migraine preventive treatments may also effectively manage VM [138]. As a result, several migraine treatments have been studied in this specific context.

##### Beta-Blockers

A controlled clinical trial with 64 patients demonstrated that propranolol reduced the intensity of both headaches and vertigo [139]. Additionally, beta-blockers are generally well tolerated and patients show good adherence to treatment. Consequently, based on the evidence available, they are recommended as a first-line option for the prophylaxis of VM. Treatment usually starts at 40 mg daily, with titration every 2–3 weeks up to a maximum of 120 mg taken twice daily [140].

##### Antidepressants

Venlafaxine, used at doses between 37.5–75 mg daily, shows effectiveness similar to propranolol and may be especially beneficial for patients with coexisting anxiety or depression. Nevertheless, venlafaxine is typically viewed as a second-line treatment for preventing VMs [139]. A multicentre study with 31 patients found that amitriptyline, administered at daily doses of 10–25 mg, reduced both the intensity and frequency of vertigo. However, even at these low doses, adverse effects are commonly reported [141].

##### Calcium Channel Blockers

Flunarizine has been suggested as a therapy for vertigo, hence its consideration for VM treatment. While evidence is still scarce, a randomised trial involving 48 patients on a 10 mg dose of flunarizine showed a notable decrease in both the frequency and severity of attacks compared with a control group that did not receive preventive treatment [142]. Notably, flunarizine has also demonstrated safety and efficacy in children, which is of relevance given the high prevalence of vestibular migraine in the paediatric population and the need for well-tolerated preventive therapies in this age group. Additional side effects encompass depression, frequently occurring alongside chronic pain or severe vestibular symptoms, as well as weight gain, which is particularly concerning given that obesity and overweight are acknowledged risk factors for the progression of migraines into chronic conditions [142].

##### Anticonvulsants

A comparative study involving 75 patients revealed that valproic acid and venlafaxine were both effective as preventive treatments for VM. Valproic acid, administered at 500 mg every 12 h, proved to be the most effective in decreasing the frequency of vertigo attacks, though it was found to be less successful in reducing the intensity of vertigo episodes [143]. Additionally, a retrospective analysis involving 19 patients who received lamotrigine (100 mg daily) noted a decrease in the frequency of vertigo. However, the reduction in vertigo and headache intensity was not statistically significant [144]. A separate study of 30 patients treated with topiramate (25–50 mg every 12 h) demonstrated improvements in the severity and frequency of both vertigo and headache symptoms [145]. While neuromodulators can be effective, their use is constrained by tolerability issues and guidelines advising against their use in women of reproductive age who do not utilise highly effective contraceptive methods because of potential teratogenic effects.

##### Acetazolamide

A retrospective study involving 50 patients found that acetazolamide (250 mg every 12 h) might effectively prevent VMs. Nevertheless, it is frequently poorly tolerated and has high discontinuation rates [146]. Acetazolamide, however, inhibits carbonic anhydrase, which alters inner ear fluid balance by modulating systemic acid–base levels. In suspected overlap syndromes involving VM and MD, this mechanism might reduce endolymphatic pressure and lessen vertigo, suggesting that it could be a valuable option in cases with overlapping clinical [93].

In summary, support for nonspecific preventive treatments remains quite limited. However, beta-blockers and antidepressants are usually viewed as first-line options because of their good tolerability and reduced risk of side effects. Similar to other types of migraines, when conventional preventives fail, botulinum toxin A and anti-CGRP therapies have been considered [147]. The relevant data are summarised in Table 3, with information mainly from non-randomised studies. The variation in the severity of vestibular symptoms was assessed using the Dizziness Disability Inventory (DHI) and the frequency of vestibular symptoms was assessed by counting defined dizziness days (DDD).

##### Botulinum Toxin Type A

A pre-post study with 20 patients showed a notable decrease in the average monthly occurrence of migraine and vertigo episodes after botulinum toxin injections under the PREEMPT protocol [148]. These results are supported by a prospective, non-randomised, controlled trial involving 60 patients. This research compared those on standard preventive therapies (propranolol, amitriptyline or flunarizine) to those who also had botulinum toxin injections. The group receiving botulinum toxin exhibited significantly better improvements in migraine-related disability scores [149]. Thus, botulinum toxin offers a promising treatment choice for VM and may be more effective in alleviating headache symptoms rather than vestibular ones [121].

##### Anti-CGRP Therapies

Anti-CGRP monoclonal antibodies, designed for treating both acute and preventive migraine episodes, have also demonstrated potential in managing VM. A randomized, prospective, double-blind, placebo-controlled clinical trial with 38 participants, comparing galcanezumab with placebo, demonstrated a reduction in the Vestibular Migraine Patient Assessment Tool and Disability Inventory (VM-PATHI) score of 5.1 points for placebo and 14.8 points for galcanezumab, demonstrating the efficacy of galcanezumab in the treatment of VM [146]. A retrospective analysis involving 21 patients who received erenumab 140 mg indicated that anti-CGRP therapies could be beneficial for individuals who do not respond to standard migraine preventive treatments [150]. A Japanese observational study with 12 patients similarly found that treatment using erenumab or galcanezumab was more effective than standard therapies in preventing episodes for those with VMs [151]. Additionally, a prospective observational cohort study involving 50 patients assessed erenumab 140 mg, fremanezumab 225 mg and galcanezumab 120 mg, showing decreases in vestibular symptoms, vertigo-related disability and headache frequency [152]. A retrospective review of 25 patients treated with anti-CGRP agents—fremanezumab 225 mg monthly, erenumab 140 mg monthly, galcanezumab 120 mg monthly or ubrogepant 100 mg daily—revealed that most patients experienced at least mild improvement in vestibular symptoms. This included eight out of nine with erenumab, seven out of nine with fremanezumab, five out of five with galcanezumab and one out of two with ubrogepant [153]. These findings are further supported by a systematic review that provided preliminary evidence of the efficacy of CGRP-targeted monoclonal antibodies in the preventive treatment of VM [154]. In conclusion, medications that target CGRP have shown recent clinical benefits for patients with VM. Although the existing evidence stems primarily from studies with limited statistical robustness, these results offer a hopeful perspective for developing effective preventive pharmacological strategies in this population.

## 8. Future Research

Significant advancements have been made in understanding VM, particularly regarding its pathophysiological mechanisms. However, there remains a pressing need for further research. Specifically, well-structured controlled trials are crucial for establishing stronger evidence for both acute and preventive treatment approaches. Moving forward, it is quite probable that CGRP-targeted therapies will become increasingly important in the clinical management of this condition.

## 9. Conclusions

VM is a common cause of episodic vertigo and poses a diagnostic challenge due to its clinical similarities with other vestibular disorders. Diagnosis relies on established clinical criteria that integrate migraine-like headaches with vestibular symptoms, with no identifiable oto-vestibular pathology present. Its pathophysiology is intricate, involving interactions between the cranial nociceptive and vestibular systems, as well as the release of neuropeptides such as CGRP.

While there have been advancements in understanding the condition, treatment options remain inadequately defined. For acute management, proposed combination strategies include using NSAIDs or triptans alongside antihistamines. In terms of prevention, the current evidence is limited and primarily relies on nonspecific migraine treatments. Among these options, beta-blockers and antidepressants seem to be the most effective. Recently, therapies targeting CGRP have displayed encouraging preliminary results, yet further high-quality studies are essential to validate their efficacy and clarify their role in clinical practice.

Considering the existing evidence gaps, a personalised treatment strategy is vital. It should combine the best available data with patient-specific factors, such as clinical presentation, demographics and comorbidities. Future randomised controlled trials are essential for developing clear, evidence-based guidelines for the acute and preventive management of vestibular issues.

## Figures and Tables

**Table 1:** (**A**) Diagnostic criteria for definite vestibular migraine [51]. ICHD-3: International Classification of Headache Disorders. (**B**) Diagnostic criteria for probable vestibular migraine [51]. VM: Vestibular migraine.

(**A**)	
**1.**	At least five episodes with moderate-to-severe vestibular symptoms, lasting 5 min to 72 h
**2.**	Current or previous history of migraine with or without aura according to the ICHD-3
**3.**	One or more migraine features with at least 50% of the vestibular episodes: Headache with at least two of the following: one-sided location, pulsating quality, moderate or severe pain intensity and aggravation by routine physical activity Photophobia and phonophobia –visual aura
**4.**	Not better accounted for by another vestibular or ICHD-3 diagnoses
(**B**)	
**1.**	At least five episodes with moderate-to-severe vestibular symptoms, lasting 5 min to 72 h
**2.**	Only one of the criteria B and C for VM is fulfilled (migraine history or migraine features during the episode)
**3.**	Not better accounted for by another vestibular or ICHD diagnosis

**Table 2:** Characteristics of the main differential diagnoses of vestibular migraine.

	Vestibular Migraine	BPPV	Menière’s Disease	Stroke/TIA	Vestibular Paroxysmia	OCD	PPPD
**Headache**	Potentially present	Possible	Possible	Potentially present	Possible	Possible	Possible
**Nystagmus**	Variable	Positional fatigable	Irritative (attack)/ paretic (recovery)	Bidirectional nystagmus/ gaze evoked	Irritative (attack)/ paretic (recovery)	Inconsistent	-
**Vertigo onset**	5 min–72 h	Seconds	20 min–12 h	Hours	Seconds	Sound/ Barometric inducement	Mostly absent
**Auditory** **findings**	Bilateral high-frequency hearing loss Aural fullness Tinnitus	-	Unilateral low-frequency hearing loss * Aural fullness Tinnitus	Abrupt/absent	Aural fullness Tinnitus	Autophony/ aural fullness	Tinnitus
**Cortical hyperexcitability symptoms**	Photophobia, phonophobia and osmophobia	-	-	-	-	-	-
**Focal** **neurological symptoms**	-	-	-	Dysarthria, dysphagia, ataxia, diplopia	-	-	-
**Phobic** **avoidance** **behaviours**	-	Present	-	-	-	-	Constant

* Hearing loss that is unilateral is present in unilateral MD, while in bilateral presentations, symptoms manifest bilaterally.

**Table 3:** Treatments for preventing VM.

Treatment	Medication	Effect Vestibular Symptoms	Comments
Nonspecific preventives	Propranolol 40–120 mg QD	Decreased frequency and intensity	Generally well tolerated
	Venlafaxine 37.5–75 mg QD	Decreased frequency and intensity	Better in patients with anxiety and depression
	Amitriptyline 10–25 mg QD	Decreased frequency and intensity	Adverse effects despite low doses
	Flunarizine 10 mg QD	Decreased frequency and intensity	Caution in the elderly due to extrapyramidal symptoms
	Valproic acid 500 mg Q 12 h	Decreased frequency without intensity reduction	Poor tolerability. Teratogenic
	Lamotrigine 100 mg QD	Decreased frequency without intensity reduction	Less effective
	Topiramate 25–50 mg Q 12 h	Decreased frequency and intensity	Frequent side effects, not for women of childbearing age
	Acetazolamide 250 mg Q 12 h	Decreased frequency and intensity	Poorly tolerated, high discontinuation rate
Botulinum toxin type A	Onabotulinum toxin A 195 UI	Decreased vertigo-related disability	More effective for headaches than for vertigo
Anti-CGRP therapy	Erenumab 140 mg monthly	Decreased vertigo-related disability	Well tolerated, few interactions or side effects
	Fremanezumab 225 mg monthly		
	Galcanezumab 120 mg monthly		
	Ubrogepant		

## Data Availability

No new data were created during this study.

## Abbreviations

CGRP	Calcitonin gene-related peptide
PPPD	Persistent postural-perceptual dizziness
